# Editorial: Collection of COVID-19 induced biases in medical research

**DOI:** 10.3389/fmed.2025.1605703

**Published:** 2025-05-19

**Authors:** Reza Rastmanesh, Vasiliki Epameinondas Georgakopoulou, Martin Wolkewitz

**Affiliations:** ^1^Department of Biomedical Sciences, American Physical Society, College Park, MD, United States; ^2^Department of Pathophysiology, Laiko General Hospital, National and Kapodistrian University of Athens, Athens, Greece; ^3^Institute of Medical Biometry and Statistics, Division Methods in Clinical Epidemiology, Faculty of Medicine and Medical Center - University of Freiburg, Freiburg, Germany

**Keywords:** COVID-19, bias, methodology, vaccination, face mask, study design

The COVID-19 pandemic has significantly impacted various aspects of social interactions, individual behaviors, and healthcare practices. It has also altered many physiological responses, leading to the expectation that numerous medical studies may be affected by hidden biases related to the pandemic, either directly or indirectly linked to the use of face masks or the virus itself. For example, wearing face masks has been shown to create substantial biases in fields such as endocrinology, ophthalmology (especially concerning dry eye and ocular conditions), sleep research, cognitive biases (including studies on emotion recognition), and gender differences, among others. It is likely that many of these biases remain unrecognized in other medical fields.

This Research Topic encompasses submissions that address previously unreported biases arising from the COVID-19 pandemic and/or the use of face masks. Our objective was to compile manuscripts that identify novel biases, thereby facilitating a more accurate and impartial interpretation of clinical findings, methodological advancements, registered clinical trials, cohort studies, and comparative studies conducted both before and after the pandemic.

It is not surprising to state that the COVID-19 lockdown significantly affected many healthcare systems. Turati et al. demonstrated that the orthopedic and trauma departments in Italy encountered major difficulties, leading to a notable decrease in all services, such as emergency consultations, outpatient visits, and surgical procedures. This situation provides important lessons for the future, but tackling a future pandemic will necessitate collaboration across multiple disciplines.

This subsequent finding may be referred to as a “policy bias” indirectly imposed by the COVID-19 pandemic.

To improve healthcare comprehensively, public health initiatives have shifted from focusing solely on pandemic response to gaining a deeper understanding of the aftermath, which includes mental health challenges arising from societal restrictions and safety measures. The lasting impacts of the COVID-19 pandemic depend on the health system's ability to foster healthier communities, enhance individual resilience, and reduce environmental stressors moving forward. In this context, the pandemic's consequences have been examined in connection with the public health crisis and the physical isolation caused by the SARS-CoV-2 virus.

Marsico and Russo state that in addition to a person's willingness to embrace positive change, the pandemic has led to emotional instability, created lasting memories, and caused social upheaval in both private and public spheres. These groups, which are often more socially disadvantaged than others, may undermine their own confrontational behavior and be less capable of demonstrating collective resilience over time.

This confrontational behavior could inadvertently exacerbate systemic biases in medical research and policy.

A key concern for the scientific community is the rate of retractions that occurred during the COVID-19 pandemic.

Furuse showed that retraction rates generally increased until at least 2019, with the highest rates observed in the category of “Neoplasms”. During the COVID-19 pandemic, there was a significant surge in publications related to “Infections” and “Respiratory Tract Diseases”; however, the retraction rates for these categories and for COVID-19-related papers were not particularly high compared to other diseases. Most disease categories showed a stronger association with retractions in China, while for COVID-19 papers, other countries exhibited higher retraction rates than China. In recent years, papers that have been retracted are less likely to appear in high-impact journals.

This phenomenon can be classified as publication bias.

Numerous research efforts have sought to assess the severity and patterns of COVID-19. Initially, during the pandemic, the complex trajectories of patients were described only in general terms, and many studies were significantly impacted by biases related to time, selection, and competing risks.

Lucke et al. demonstrated that multi-state models help mitigate these biases by simultaneously analyzing various clinical outcomes while considering their time-related nature, including ongoing cases, and accounting for competing events. A group of researchers utilized a publicly accessible dataset from COVID-19 first wave to illustrate the advantages of employing multi-state methodology in the analysis of hospital data.

They evaluated the results of the data analysis conducted with multi-state models against the results obtained when different types of bias were overlooked. Additionally, Cox regression was employed to analyze the transitions between states in the multi-state model, enabling a comparison of how covariates affect transition rates between the two states. Finally, they computed the anticipated lengths of stay and state probabilities derived from the multi-state model and represented this information through stacked probability plots. Utilizing multi-state models on real-time data enables quick identification of changes in disease progression when new variants emerge. This information is crucial for guiding medical and political leaders, as well as the general public.

Another three common methodological biases need to be addressed: competing risks, immortal-time bias, and confounding bias in real-world observational studies that assess treatment effectiveness. A team of researchers utilized a specific observational data example involving COVID-19 patients to evaluate the effects of these biases and suggest possible solutions. Indeed, neglecting competing risks, immortal-time bias, and confounding bias can distort treatment effect estimates.

According to Martinuka et al., using the basic Kaplan-Meier method produced the most inaccurate results, leading to inflated probabilities for the primary outcome in studies involving COVID-19 hospital data. This inflation could misguide clinical decisions. Therefore, it is essential to tackle both immortal-time bias and confounding bias when evaluating treatment effectiveness. The trial emulation framework presents a possible approach to mitigate all three of these methodological biases.

This was only a part of the issue. Tackling bias in how SARS-CoV-2 reinfection is defined is another key challenge. Traditionally, reinfection is identified as a positive test result that happens at least 90 days after a prior infection has been diagnosed. However, this lengthy timeframe might result in an undercount of reinfection cases. Chemaitelly et al. explored the possibility of using a different, shorter timeframe to define reinfection. The 40-day time window was appropriate for defining reinfection, irrespective of whether it was the first, second, third, or fourth occurrence. The sensitivity analysis, confined to high testers exclusively, replicated similar patterns and results. These findings will significantly impact the issue of underestimation.

The comparison between immunity gained from previous natural infections and that obtained through vaccination against SARS-CoV-2 is a significant topic. In this context, we required a statistical clarification to prevent any misinterpretation. To achieve this goal, we need access to real-world data from a large population. Weber et al. analyzed data from over 52,000 individuals. The group that was infected tended to be younger, had a higher proportion of men, and exhibited lower morbidity compared to the vaccinated group. After the initial 90 days, these differences became more pronounced. The analysis conducted during the second 90 days revealed variations in results based on different analytical methods and age groups. There were also age-related differences in mortality rates. When considering the outcome of SARS-CoV-2 infection, the impact of vaccination compared to infection differs by age, showing a disadvantage for vaccinated individuals in the younger demographic, while no significant difference was observed in older adults. It is important to analyze two observation periods: the first and second 90-day spans after infection or vaccination. Furthermore, it is necessary to implement methods to correct any imbalances. This strategy facilitates equitable comparisons, enables more thorough conclusions, and helps avoid biased interpretations. It is crucial not to mix up these results with the 40-day time frame that was proposed as suitable for identifying reinfection (Chemaitelly et al.).

As for the observational studies on the effectiveness of COVID-19 vaccines, these designs have provided crucial real-world insights that have influenced global public health policies. These studies, which mainly utilize existing data sources, have been crucial for evaluating vaccine effectiveness across various populations and for creating effective vaccination strategies. Cohort designs are commonly used in this research. The swift rollout of vaccination campaigns during the pandemic led to variations in vaccination rates influenced by socio-demographic factors, public policies, perceived risks, health-promoting behaviors, and overall health status. This may have resulted in biases such as healthy user bias, healthy vaccine effect, frailty bias, differential depletion of susceptibility bias, and confounding by indication. The pressure to publish findings rapidly may have exacerbated these biases or led to their oversight, thereby affecting the reliability of the results. The extent of these biases can vary greatly depending on the context, data sources, and analytical techniques used, and they are likely to be more pronounced in low- and middle-income countries due to weaker data infrastructure. It is crucial to address and reduce these biases to obtain accurate estimates of vaccine effectiveness, inform public health strategies, and maintain public confidence in vaccination efforts. Agampodi et al. in their brilliant article state that clear communication about these biases and a commitment to improving the design of future observational studies are vital.

Another type of neglected bias that may obscure data analysis during the COVID-19 pandemic arises from treatment-induced differences. Prosty et al. demonstrated that during the pandemic, many patients received concomitant corticosteroids, which are known to broadly suppress inflammatory cytokines, including those associated with type II inflammation. This may have obscured any differences induced by omalizumab and biased the results toward the null hypothesis, while others did not receive corticosteroid therapy. Results from one of the articles submitted to our Research Topic suggested that the potential benefits of omalizumab in COVID-19 may be mediated independently of the modulation of the measured serum biomarkers. This finding, in itself, impacts the interpretation of many clinical trials conducted during the pandemic.

Given the numerous issues addressed in this brief editorial, the significance of interdisciplinary collaboration in mitigating biases exacerbated by the pandemic must be emphasized.

